# HIV-1 gp120 and Modified Vaccinia Virus Ankara (MVA) gp140 Boost Immunogens Increase Immunogenicity of a DNA/MVA HIV-1 Vaccine

**DOI:** 10.1128/JVI.01077-17

**Published:** 2017-11-30

**Authors:** Xiaoying Shen, Rahul Basu, Sheetal Sawant, David Beaumont, Sue Fen Kwa, Celia LaBranche, Kelly E. Seaton, Nicole L. Yates, David C. Montefiori, Guido Ferrari, Linda S. Wyatt, Bernard Moss, S. Munir Alam, Barton F. Haynes, Georgia D. Tomaras, Harriet L. Robinson

**Affiliations:** a Duke Human Vaccine Institute, Department of Medicine, Duke University School of Medicine, Durham, North Carolina, USA; b GeoVax, Inc., Smyrna, Georgia, USA; c Department of Surgery, Duke University Medical Center, Durham, North Carolina, USA; d Laboratory of Viral Diseases, National Institute of Allergy and Infectious Diseases, National Institutes of Health, Bethesda, Maryland, USA; e Department of Immunology, Duke University Medical Center, Durham, North Carolina, USA; f Department of Molecular Genetics and Microbiology, Duke University Medical Center, Durham, North Carolina, USA; Emory University

**Keywords:** DNA/MVA, HIV vaccine, T cell response, antibody response, boost immunogen, nonhuman primate

## Abstract

An important goal of human immunodeficiency virus (HIV) vaccine design is identification of strategies that elicit effective antiviral humoral immunity. One novel approach comprises priming with DNA and boosting with modified vaccinia virus Ankara (MVA) expressing HIV-1 Env on virus-like particles. In this study, we evaluated whether the addition of a gp120 protein in alum or MVA-expressed secreted gp140 (MVAgp140) could improve immunogenicity of a DNA prime-MVA boost vaccine. Five rhesus macaques per group received two DNA primes at weeks 0 and 8 followed by three MVA boosts (with or without additional protein or MVAgp140) at weeks 18, 26, and 40. Both boost immunogens enhanced the breadth of HIV-1 gp120 and V1V2 responses, antibody-dependent cellular cytotoxicity (ADCC), and low-titer tier 1B and tier 2 neutralizing antibody responses. However, there were differences in antibody kinetics, linear epitope specificity, and CD4 T cell responses between the groups. The gp120 protein boost elicited earlier and higher peak responses, whereas the MVAgp140 boost resulted in improved antibody durability and comparable peak responses after the final immunization. Linear V3 specific IgG responses were particularly enhanced by the gp120 boost, whereas the MVAgp140 boost also enhanced responses to linear C5 and C2.2 epitopes. Interestingly, gp120, but not the MVAgp140 boost, increased peak CD4^+^ T cell responses. Thus, both gp120 and MVAgp140 can augment potential protection of a DNA/MVA vaccine by enhancing gp120 and V1/V2 antibody responses, whereas potential protection by gp120, but not MVAgp140 boosts, may be further impacted by increased CD4^+^ T cell responses.

**IMPORTANCE** Prior immune correlate analyses with humans and nonhuman primates revealed the importance of antibody responses in preventing HIV-1 infection. A DNA prime-modified vaccinia virus Ankara (MVA) boost vaccine has proven to be potent in eliciting antibody responses. Here we explore the ability of boosts with recombinant gp120 protein or MVA-expressed gp140 to enhance antibody responses elicited by the GOVX-B11 DNA prime-MVA boost vaccine. We found that both types of immunogen boosts enhanced potentially protective antibody responses, whereas the gp120 protein boosts also increased CD4^+^ T cell responses. Our data provide important information for HIV vaccine designs that aim for effective and balanced humoral and T cell responses.

## INTRODUCTION

GOVX-B11, a subtype B DNA prime-modified vaccinia virus Ankara (MVA) boost vaccine, elicits higher response rates and titers of antibodies to the gp41 transmembrane than the gp120 receptor binding subunit of Env (1). Both components of GOVX-B11 express virus-like particles (VLPs) displaying membrane-bound Env. The current study was undertaken to test the ability of gp120 protein or MVA-expressed secreted gp140 (MVAgp140; the ectodomain of Env) to boost the ability of GOVX-B11 to elicit gp120 antibodies. Increasing the titers of antibodies to gp120 and, in particular, to the V1V2 region of gp120 is considered desirable because gp120 is the major target for neutralizing antibodies for HIV (2) and because binding antibodies for V1V2 were a correlate for reduced HIV-1 risk in the partially efficacious RV144 vaccine trial in Thailand (3, 4).

The GOVX-B11 vaccine expresses the native ADA (clade B) gp160 Env, a clade B CCR-5-tropic Env from a chronic infection (5). For the gp120 boost, B.63521Δ11mutC, a clade B transmitted founder (T/F) Env, was chosen because of its favorable antigenicity and immunogenicity (6) and its projected availability as a current good manufacturing practices (cGMP) product. B.63521Δ11mutC has an 11-amino-acid (aa) truncation at the N terminus of gp120 that enhances antigenicity of the V2 and C1 epitopes that are targets for antibody-dependent cell-mediated cytotoxicity (ADCC) (7, 8) and prevents gp120 dimerization (8), thus optimizing manufacture. B.63521Δ11mutC also has a mutated V3 loop to prevent clipping of gp120 when expressed in CHO cells for manufacturing (S. M. Alam, H. X. Liao, and B. F. Haynes, unpublished data). Alum (Alhydrogel) was chosen as the adjuvant for the B.63521Δ11mutC protein boost because of its use in the RV144 HIV-1 vaccine phase 2b trial that demonstrated an estimated 31.2% vaccine efficacy (9). ADA gp140 was used for the MVA-expressed gp140 boost because this gp140 shows unusual stability as a secreted gp140 (B. Moss, personal communication).

The regimen for vaccination was based on the regimen used in RV144 and on our clinical experience with DNA priming and MVA boosting (1, 9–11). The RV144 regimen codelivered gp120 protein boosts with the ALVAC canarypox vaccine used for both priming and boosting. ALVAC was delivered to one arm and gp120 protein to the opposite arm. In this study, we delivered the gp120 protein or MVAgp140 boosts to the contralateral arm from the MVA62B boosts and used our most successful clinical regimen for immunizations: priming with DNA at months 0 and 2 and boosting with MVA at months 4, 6, and 10 (11).

Following the 3rd boost, the groups with gp120 and MVAgp140 in boosts had comparable increases in gp120 and V1V2 binding antibody responses, whereas the plus-gp120 group had enhanced CD4^+^ T cells over the MVA-only group. Given the comparable levels of elicited antibodies, and the potential for vaccine-elicited CD4^+^ T cells both to protect from infection (12) and to serve as targets for infection (13, 14), MVAgp140 and the more generally used gp120 boost immunogens merit further evaluation for advancement to clinical studies.

## RESULTS

### Serum binding antibody response.

**FIG 1:**
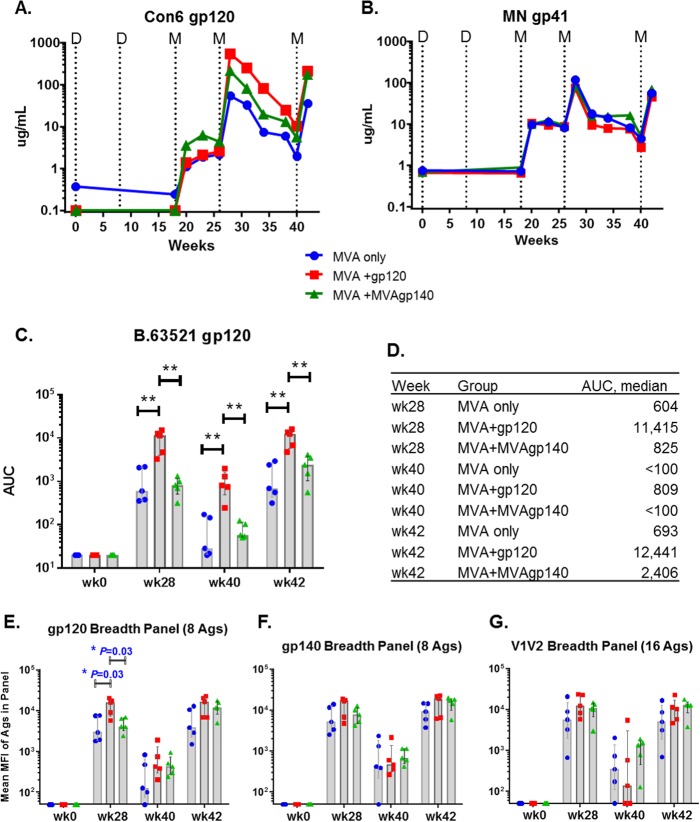
Magnitude and breadth of binding antibody responses. (A and B) Group median IgG binding magnitude for Con6 gp120 (A) and MN gp41 (B) of longitudinal sera measured in ELISA. Dotted lines indicate time of immunizations. D, DNA prime at weeks 0 and 8; M, MVA boost at weeks 18, 26, and 40. (C and D) Binding AUC (measured in BAMA) for individual animals (C) and for group medians (D) to B.63521 gp120. (E to G) Mean MFI for binding to the gp120 (C), gp140 (D), or V1V2 (E) breadth panel measured in BAMA. MFIs for gp120 and gp140 antigens were obtained from 1:400 plasma dilutions. MFIs for V1V2 antigens were obtained from 1:80 plasma dilutions. Each symbol represents binding by one plasma sample to one antigen panel. Shaded bars represent group median values and error bars represent interquartile ranges. Group median values are also listed in Table 1. *, *P* < 0.05 for pairwise comparison (exact Wilcoxon rank sum; values shown in Table 2). All differences are insignificant once adjusted for multiple comparison (Table 2).

Binding antibody responses against a consensus group M gp120 (Con6 gp120) and MN gp41 were measured using enzyme-linked immunosorbent assays (ELISAs) and longitudinal serum samples. gp120- and gp41-specific IgG responses developed 2 weeks after the first MVA boost (week 20), peaked after the second boost (week 28) declined with time, and were boosted again after the 3rd boost (Fig. 1A and B). At 2 weeks after the 2nd boost (week 28), the group treated with MVA plus gp120 (MVA+gp120 group) elicited the highest levels of Con6 gp120-specific binding response, with a median titer 10-fold higher than that of the MVA-only group and 2.5-fold higher than that of the MVA+MVAgp140 group (Fig. 1A). Following the 3rd boost, the MVA+gp120 and MVA+MVAgp140 groups had similar median magnitudes of antibody responses, whereas the titer elicited by MVA only was 4-fold lower. However, the differences among groups were not significant (Fig. 1A). Binding magnitudes for MN gp41 were overall comparable among groups at all time points tested (Fig. 1B).

Next, we measured IgG responses to the vaccine-matched B.63521 gp120 protein. The MVA+gp120 group showed significantly higher responses following the 2nd boost, postcontraction at the time of the 3rd boost, and after the 3rd boost than did both the MVA-only and MVA+MVAgp140 groups (Fig. 1C). The differences between the MVA+gp120 group and the MVA+MVAgp140 group were 13.8-fold and 5.2-fold at weeks 28 and 42, respectively (Fig. 1D).

### Breadth of binding antibody response.

Following the 3rd boost, the MVA+gp120 and MVA+MVAgp140 groups had similar magnitudes and breadths of binding antibodies for reference panels of gp120 and gp140 antigens (Fig. 1E and F and Table 1). However, transient differences in magnitudes and breadths were seen after the 2nd boost for gp120 and gp140, with the group median binding mean fluorescence intensity (MFI) of the MVA+gp120 group being significantly higher for the gp120 antigen panel (*P* values that are not controlled for multiple comparison [raw_p] = 0.03 for each) (Fig. 1E and Tables 1 and 2) and trending higher for the gp140 panel (raw_p > 0.05) (Fig. 1F and Tables 1 and 2) than the other two groups.

**TABLE 1:** Magnitude for binding to antigen panels in Fig. 1C to E, fold increase over the MVA-only group, and fold contraction and boost^*a*^

Antigen panel	Group	Group median binding magnitude:			Fold difference	
		Wk 28	Wk 40	Wk 42	Contraction (wk 28/wk 40)	Boosting (wk 42/wk 28)
gp120 breadth (8 Ags)	MVA only	2,963	127	3,895	15.7	1.4
	+gp120	15,914	427	16,660	13.7	1.1
	+MVA gp140	4,060	427	11,855	8.1	2.6
gp140 breadth (8 Ags)	MVA only	5,215	424	9,223	8.7	1.5
	+gp120	16,799	466	19,009	17.7	1.2
	+MVA gp140	7,794	680	17,323	8.8	1.8
V1V2 breadth (16 Ags)	MVA only	5,670	350	5,026	13.3	1.0
	+gp120	12,308	136	10,477	42.6	0.9
	+MVA gp140	10,808	1,347	12,685	8.0	1.2

a Magnitude of binding to each antigen panel was calculated as mean MFI for each breadth antigen panel as described for Fig. 1E–1G. Fold of contraction and Fold of boost were calculated as described for Fig. 2A and 2B. Values shown are group medians. Fold over MVA-only group was calculated as group median of MVA+gp120 or MVA+MVAgp140 group divided by that of the MVA-only group, for each antigen panel.

**TABLE 2:** Raw *P* values and FDR-corrected (BH method) *P* values for pairwise comparisons between groups

Comparison group and wk^*a*^	Parameter^*b*^	Raw_p^*c*^	fdr_p^*d*^	Figure
mva vs gp140, wk 28	BAMA, agg.gp120, mean MFI	0.841	0.993	1E
mva vs gp120, wk 28	BAMA, agg.gp120, mean MFI	0.032	0.185	1E
gp120 vs gp140, wk 28	BAMA, agg.gp120, mean MFI	0.032	0.185	1E
mva vs gp140, wk 42/28	Boost fold, agg.V1V2, MFI ratio	0.056	0.275	2
mva vs gp140, wk 42/28	Boost fold, agg.gp120, MFI ratio	0.016	0.140	2
mva vs gp140, wk 42/28	Boost fold, agg.gp140, MFI ratio	0.151	0.506	2
mva vs gp120, wk 42/28	Boost fold, agg.V1V2, MFI ratio	0.421	0.793	2
mva vs gp120, wk 42/28	Boost fold, agg.gp120, MFI ratio	0.421	0.793	2
mva vs gp120, wk 42/28	Boost fold, agg.gp140, MFI ratio	0.222	0.611	2
gp120 vs gp140, wk 42/28	Boost fold, agg.V1V2, MFI ratio	0.032	0.185	2
gp120 vs gp140, wk 42/28	Boost fold, agg.gp120, MFI ratio	**0.008**	0.100	2
gp120 vs gp140, wk 42/28	Boost fold, agg.gp140, MFI ratio	**0.008**	0.100	2
mva vs gp140, wk 28	Neut, 25710, ID_50_ titer	1.000	1.000	5F
mva vs gp120, wk 28	Neut, 25710, ID_50_ titer	0.040	0.220	5F
gp120 vs gp140, wk 28	Neut, 25710, ID_50_ titer	0.040	0.220	5F
mva vs gp140, wk 28	ADCC, B.63521, peak activity	0.222	0.611	5I
mva vs gp140, wk 42	ADCC, B.63521, peak activity	0.151	0.506	5I
mva vs gp140, wk 28	ADCC, B.63521, titer	0.333	0.708	5J
mva vs gp140, wk 42	ADCC, B.63521, titer	0.016	0.140	5J
mva vs gp120, wk 28	ADCC, B.63521, peak activity	0.032	0.185	5I
mva vs gp120, wk 42	ADCC, B.63521, peak activity	**0.008**	0.100	5I
mva vs gp120, wk 28	ADCC, B.63521, titer	0.016	0.140	5J
mva vs gp120, wk 42	ADCC, B.63521, titer	**0.008**	0.100	5J
gp120 vs gp140, wk 28	ADCC, B.63521, peak activity	0.016	0.140	5I
gp120 vs gp140, wk 42	ADCC, B.63521, peak activity	**0.008**	0.100	5I
gp120 vs gp140, wk 28	ADCC, B.63521, titer	0.016	0.140	5J
gp120 vs gp140, wk 42	ADCC, B.63521, titer	**0.008**	0.100	5J
mva vs gp140, wk41	ICS, PBMC, CD4, IL-2, proportion	1.000	1.000	7A
mva vs gp140, wk41	ICS, PBMC, CD4, TNF-a, proportion	0.341	0.713	7A
mva vs gp120, wk41	ICS, PBMC, CD4, IL-2, proportion	0.032	0.185	7A
mva vs gp120, wk41	ICS, PBMC, CD4, TNF-a, proportion	0.056	0.275	7A
gp120 vs gp140, wk41	ICS, PBMC, CD4, IL-2, proportion	0.016	0.140	7A
gp120 vs gp140, wk41	ICS, PBMC, CD4, TNF-a, proportion	0.032	0.185	7A
mva vs gp140, wk 28	BAMA, B.63521, AUC	1.000	1.000	1C
mva vs gp140, wk40	BAMA, B.63521, AUC	0.690	0.937	1C
mva vs gp140, wk 42	BAMA, B.63521, AUC	0.310	0.695	1C
mva vs gp120, wk 28	BAMA, B.63521, AUC	**0.008**	0.100	1C
mva vs gp120, wk40	BAMA, B.63521, AUC	**0.008**	0.100	1C
mva vs gp120, wk 42	BAMA, B.63521, AUC	**0.008**	0.100	1C
gp120 vs gp140, wk 28	BAMA, B.63521, AUC	**0.008**	0.100	1C
gp120 vs gp140, wk40	BAMA, B.63521, AUC	**0.008**	0.100	1C
gp120 vs gp140, wk 42	BAMA, B.63521, AUC	**0.008**	0.100	1C
mva vs gp140, wk 28	Linear mapping, C2.2, signal intensity	0.032	0.185	3C
mva vs gp120, wk 28	Linear mapping, C2.2, signal intensity	0.690	0.937	3C
gp120 vs gp140, wk 28	Linear mapping, C2.2, signal intensity	0.056	0.275	3C
mva vs gp140, wk 28	Linear mapping, C2.3, signal intensity	0.690	0.937	3C
mva vs gp120, wk 28	Linear mapping, C2.3, signal intensity	0.032	0.185	3C
gp120 vs gp140, wk 28	Linear mapping, C2.3, signal intensity	0.056	0.275	3C
mva vs gp140, wk 28	Linear mapping, C5.1, signal intensity	**0.008**	0.100	3C
mva vs gp120, wk 28	Linear mapping, C5.1, signal intensity	0.841	0.993	3C
gp120 vs gp140, wk 28	Linear mapping, C5.1, signal intensity	**0.008**	0.100	3C
mva vs gp140, wk 28	Linear mapping, C5.2, signal intensity	**0.008**	0.100	3C
mva vs gp120, wk 28	Linear mapping, C5.2, signal intensity	0.722	0.945	3C
gp120 vs gp140, wk 28	Linear mapping, C5.2, signal intensity	0.048	0.260	3C
mva vs gp140, wk 42	Linear mapping, C2.1, signal intensity	0.095	0.404	3C
mva vs gp120, wk 42	Linear mapping, C2.1, signal intensity	0.222	0.611	3C
gp120 vs gp140, wk 42	Linear mapping, C2.1, signal intensity	0.032	0.185	3C
mva vs gp140, wk 42	Linear mapping, C2.2, signal intensity	**0.008**	0.100	3C
mva vs gp120, wk 42	Linear mapping, C2.2, signal intensity	**0.008**	0.100	3C
gp120 vs gp140, wk 42	Linear mapping, C2.2, signal intensity	0.095	0.404	3C
mva vs gp140, wk 42	Linear mapping, C4.2, signal intensity	0.016	0.140	3C
mva vs gp120, wk 42	Linear mapping, C4.2, signal intensity	0.722	0.945	3C
gp120 vs gp140, wk 42	Linear mapping, C4.2, signal intensity	0.016	0.140	3C

a Group abbreviations: mva, MVA-only; gp120, MVA+gp120; gp140, MVA+MVAgp140.

b agg., aggregate (mean value for each antigen panel).

c Raw *P* values are from Wilcoxon rank sum test. Only parameters that showed a *P* value of <0.05 in the omnibus test are included in the pairwise comparison and subsequent FDR correction. Underlining indicates raw_p values of <0.05, and bold and underlining indicates raw_p values of <0.01.

d FDR_p values were obtained through multiple comparison correction of 63 pairwise comparisons in this table and 264 spearman correlation tests in Table 4 (across a total of 327 tests).

In contrast to the gp120 and gp140 responses, levels of binding to the V1V2 panel were similar for sera from the MVA+gp120 and MVA+MVAgp140 groups after both the 2nd and 3rd boosts, with both being approximately 2-fold higher than that for the MVA-only group (Fig. 1G and Table 1).

The durability and boostability of binding antibody responses were evaluated as the fold decline of the antibody response over the 12 weeks following the 2nd boost (week 28/week 40) and the fold increase of the antibody response following the 3rd boost over that following the 2nd boost (week 42/week 40) (Fig. 2 and Table 1). The MVA+gp120-boosted group underwent the largest declines (Fig. 2A and Table 1). By the 3rd boost, the MVA+MVAgp140 group showed the strongest boost for all 3 antigen panels (raw_p values of 0.032 to 0.008 against the MVA+gp120 group) (Fig. 2B and Table 2).

**FIG 2:**
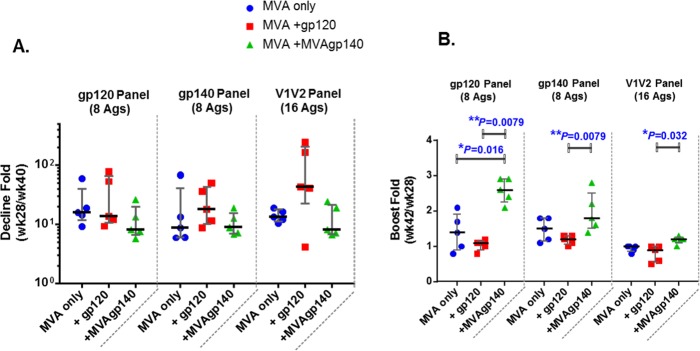
Contraction (A) and fold boost (B) of binding response for 3 antigen panels. Contraction was calculated as week 28/week 40 mean MFI for binding to the antigen panel. Fold boost was calculated as week 42/week 28 mean MFI for binding to the antigen panel. Each symbol represents the calculated ratio for each sample. Error bars represent group medians and interquartile ranges. *, *P* < 0.05, and **, *P* < 0.01, for pairwise comparison (exact Wilcoxon rank sum; values shown in Table 2). All differences are insignificant once adjusted for multiple comparison (Table 2).

### Specificity of binding antibodies for linear epitopes.

**FIG 3:**
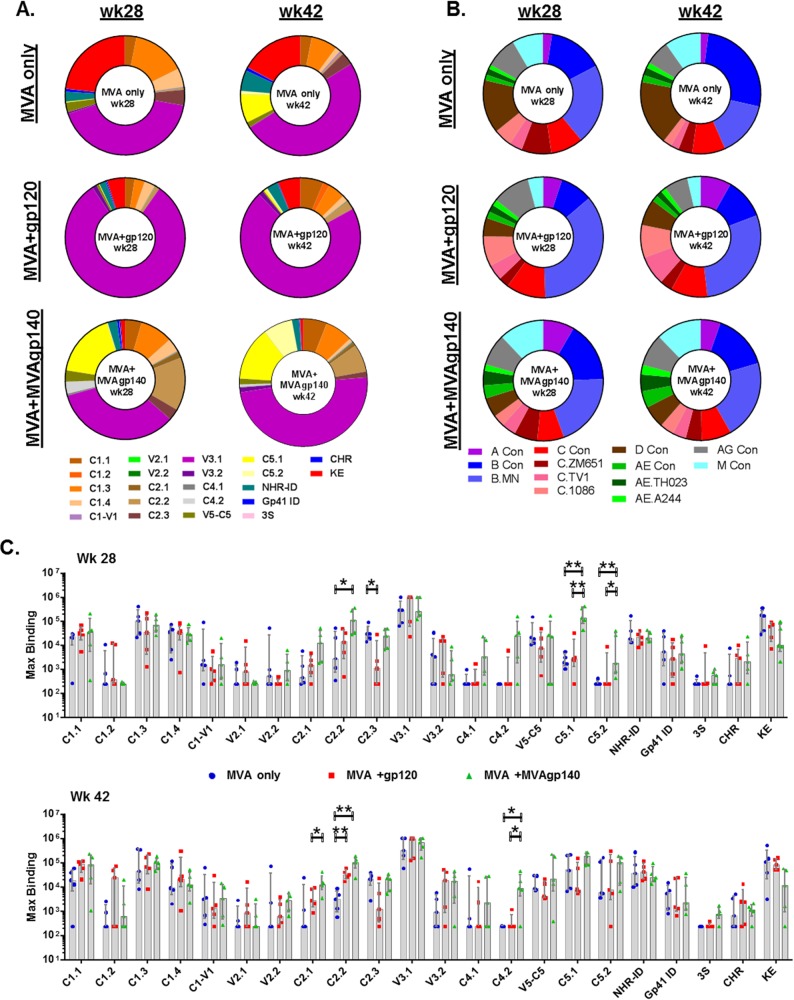
Proportions of total linear binding to each identified epitope (A) or to each strain included in the epitope mapping peptide library (B) and magnitude of binding to each epitope identified (C). Each sector in panel A represents a group median magnitude to an epitope, which was calculated as the highest binding to a single peptide within each epitope region as defined in Table 3. Each sector in panel B represents a group median total linear epitope binding for a strain, which was calculated as the sum of binding intensities to all linear peptides for that strain. Values in panel C are the highest binding to a single peptide within each epitope region. Bars represent group medians and whiskers represent interquartile ranges. *, raw_p < 0.05; **, raw_p < 0.01 (Table 2).

IgG from the vaccinated animals targeted linear epitopes in the C1, V2, C2, V3, C4, and C5 regions of gp120 (Fig. 3A and C and Table 3). In gp41, antibody responses were targeted to the C terminus of the N-heptad repeat, the N terminus of the gp41 immunodominant region (NHR_ID), the gp41 immunodominant region (gp41_ID), 3S (an epitope C terminal to gp41_ID that contains 3 serine residues), C-heptad repeat (CHR), and KE (Kennedy epitope) (Fig. 3A and C and Table 3).

**TABLE 3:** Definitions of linear epitopes identified

Epitope	Peptide region^*a*^	HXB2 amino acid no.^*b*^
C1.1	24–26	71–91
C1.2	28	83–97
C1.3	33	98–112
C1.4	35–36	104–121
C1-V1	40–42	119–139
V2.1	51–52	157–174
V2.2	59–61	181–201
C2.1	63–65	193–213
C2.2	66–68	202–222
C2.3	83–85	253–273
V3.1	98–101	298–321
V3.2	102	312–326
C4.1	137–138	427–444
C4.2	141	439–453
V5-C5	147–150	457–480
C5.1	157–159	487–507
C5.2	160–162	496–516
NHR-ID	186–190	574–600
gp41_ID	192–195	592–615
3S	198	610–624
CHR	204–205	628–645
KE	232–238	712–744

a Regions for each linear epitope identified defined as in peptide numbers in the microarray library.

b Regions for each linear epitope identified defined as amino acid numbers according to HXB2 envelope sequence. The range covers from the first amino acid of the first peptide within the epitope region to the last amino acid of the last peptide within the epitope region.

The contribution of each epitope to the total Env linear epitope binding after the 2nd and 3rd boosts was evaluated by determining the percentage of the binding intensity for each epitope within the total Env linear epitope binding intensity. Among the 3 groups, the MVA+gp120 group was the most focused on V3, with 81.1% and 70.8% of total linear epitope binding (group median) targeting V3 at weeks 28 and 42, respectively, compared to 34.2% to 49.9% for other groups (Fig. 3A). The MVA-only group showed a trend for higher proportions of binding responses targeting KE, with 23.2% and 17.1% of total linear epitope binding intensities targeting KE at weeks 28 and 42, respectively, compared to 0.8% to 6.1% for other groups (Fig. 3A). The MVA+MVAgp140 group showed a trend for higher proportions of binding responses targeting C5 and C2 (epitope C2.2) than those of the other groups (Fig. 3A).

Significant differences (raw_p < 0.05, exact Wilcoxon rank sum test) were observed among groups for binding to epitopes in C2, C4, and C5 following either the 2nd (week 28) or 3rd (week 42) boost (Fig. 3C). Highly significant differences (raw *P* < 0.01) were found after the 3rd boost for C2.2 (MVA+MVAgp140 and MVA+gp120 > MVA only) and after the 2nd boost for C5.1 (MVA+MVAgp140 > MVA only and MVA+gp120) and C5.2 (MVA+MVAgp140 > MVA only) (Fig. 3C and Table 2). Due to the large number of samples that were saturated for binding to V3.1, especially for the MVA+gp120 group, differences among groups for V3.1 binding were not evaluated.

Binding responses to peptides representing different clades/strains were generally similar among vaccine groups and at the two tested time points (following the 2nd and 3rd boosts) (Fig. 3B). All groups bound most strongly to clade B sequences. The MVA+gp120 group had slightly higher proportions of binding to clade C sequences than the other 2 groups, with binding to clade C sequences (including consensus C, C.ZM651, C.TV1, and C.1086) accounting for 25.8% and 29.9% of total linear binding at weeks 28 and 42, respectively, compared to 17.2% to 25.3% for other groups. The MVA+MVAgp140 groups showed the most “balanced” coverage of different clades, with 12 strains (out of 13 total) each contributing 3% or more to total Env binding for both time points, compared to 7 strains and 9 strains for MVA-only and MVA+gp120 groups, respectively (Fig. 3B). In particular, binding for clade AE strains (including consensus AE, AE.A244, and AE.TH023) contributed 9.1% and 11.6% to total binding for the MVA+MVAgp140 group at weeks 28 and 42, compared to 4.4% to 5.5% for the other groups.

### Avidity of antibody responses.

**FIG 4:**
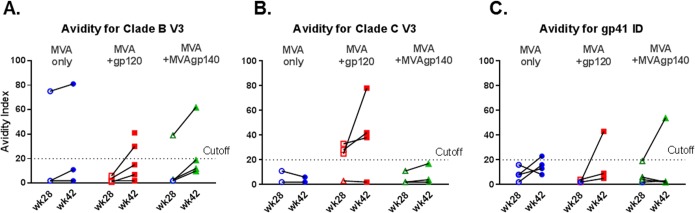
Plasma IgG avidity index from vaccinated animals for binding to clade B (A) and clade C (B) V3 and gp41_ID (C) peptides at week 28 (open symbols) and week 42 (filled symbols), measured in a BAMA-based avidity index assay. Blue, MVA-only group; red, MVA+gp120 group; green, MVA+MVAgp140 group.

The avidity of the antibody elicited for three linear epitopes—clade B V3, clade C V3, and gp41–ID—was measured by an avidity index assay (11, 15). For all animals/epitopes where the avidity indices were quantifiable, avidity indices were higher after the 3rd than after the 2nd boost (Fig. 4).

### Neutralizing antibody responses.

**FIG 5:**
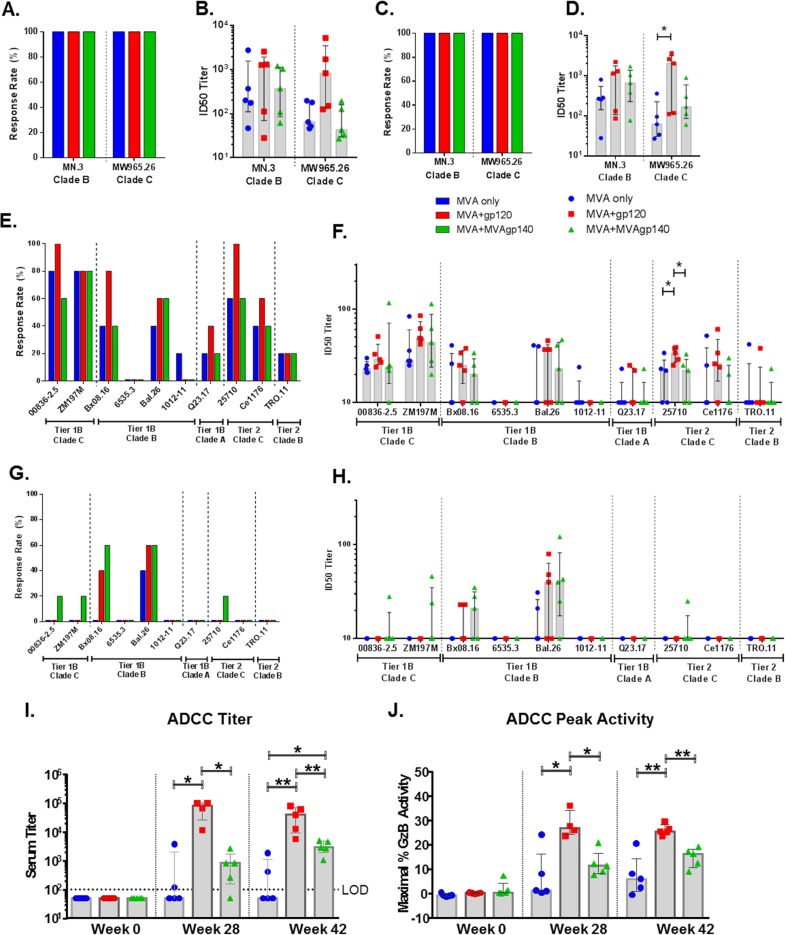
Positivity rates and ID_50_ titers of serum neutralization against tier 1A (A to D) and tier 1B and tier 2 (E to H) viruses measured in TZM-bl cells and serum titer (I) and peak granzyme B activity (J) of ADCC activity measured with B63531 gp120-coated target cells. Shaded bars represent group medians, and error bars represent interquartile ranges in panels B, D, F, and H. *, *P* < 0.05, and **, *P* < 0.01, for pairwise comparison (exact Wilcoxon rank sum test; values shown in Table 2). All differences are insignificant once adjusted for multiple comparison (Table 2).

Sera from the 3 groups neutralized tier 1A, tier 1B, and some tier 2 viruses. Animals from all vaccine groups developed neutralizing antibodies against tier 1 MN.3 (clade B) and MW965.26 (clade C) viruses, with 50% inhibitory dose (ID_50_) titers up to 5,274 (Fig. 5A to D). The MVA+gp120 group showed the highest titers after both the 2nd and 3rd boosts, and the difference was significant between MVA+gp120 and MVA-only groups after the 3rd boost for MW965.26 (raw_p = 0.03, exact Wilcoxon rank sum test; Fig. 5D and Table 2).

A subset of vaccinated animals developed neutralizing antibodies against tier 1B viruses, with titers up to 122 at week 28 (Fig. 5E and F). The MVA+gp120 group trended higher for magnitudes of neutralizing responses against both clade C and non-clade C tier 1B viruses than the other 2 groups. Except for Bal, the response rates and titers were lower for the tier 1B viruses after the 3rd than after the 2nd MVA boost (Fig. 5G and H). This contrasted with the neutralizing activity for the tier 1A viruses, which remained at similar positivity rates and titers at weeks 28 and 42 for all 3 groups. This decline in activity was particularly evident for the 2 tested clade C tier 1B viruses. After the 2nd MVA boost, response rates to both clade C tier 1B viruses were 60% or greater and several titers were above 1:80. After the 3rd MVA boost, the highest response rate was 20% and titers were below, or close to, background (1:20). The MVA+MVAgp140 group maintained low-level responses slightly better than the other 2 groups (Fig. 5G and H). Neutralization for clade B tier 1B virus BX08.16 and clade A tier 1B virus Q23.17 also declined for both positivity rates and serum titers, especially for the MVA-only and MVA+gp120 groups (Fig. 5E to H).

Notably, vaccinated animals developed low levels of neutralizing antibodies (ID_50_ titers up to 61) against tier 2 viruses after the 2nd boost, with positivity rates ranging from 40% to 100% for 2 clade C tier 2 viruses (25710-2.43 and Ce1176_A3) and being 20% for the clade B tier 2 virus TRO.11 (Fig. 5E and F). The MVA+gp120 group showed the highest positivity rate and titer among the 3 groups, with titers significantly higher than for the other 2 groups for isolate 25710-2.43 (raw_p < 0.05, exact Wilcoxon rank sum test [Table 2]). Neutralization against tier 2 viruses was undetectable following the 3rd boost for all but one animal in the MVA+MVAgp140 group (Fig. 5G and H).

### ADCC responses.

In assays using B.63521Δ11 gp120-coated target cells, ADCC activity was detected in 100% of animals in both the MVA+gp120 and MVA+MVAgp140 groups (Fig. 5I and J). Consistent with its high titers of elicited binding response for B.63521Δ11, the MVA+gp120 group showed higher ADCC titers and peak granzyme B activity than the other 2 groups (raw_p < 0.05 at week 28 and *P* < 0.01 at week 42, exact Wilcoxon rank sum test) (Fig. 5I and J and Table 2). Group median titers were 1:85,000 and 1:800 for the MVA+gp120 and MVA+MVAgp140 groups, respectively, after the 2nd boost, and 1:40,000 and 1:3,000, respectively, after the 3rd boost.

### Correlations between antibody specificities and antiviral functions.

**FIG 6:**
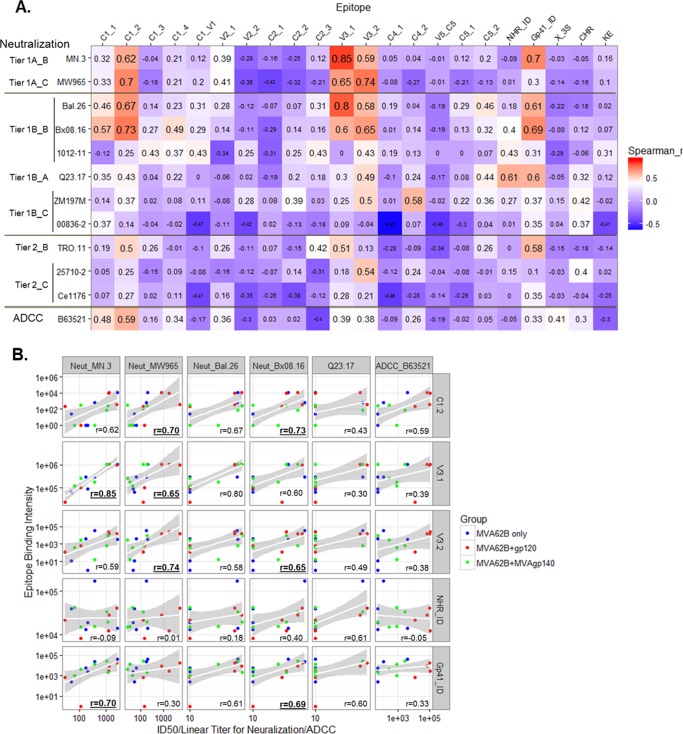
(A) Heat map of *r* values for Spearman correlation analyses between neutralization ID_50_ and ADCC titers and binding for each linear epitope; (B) scatter plots showing parameters with *r* values of >0.6 in at least one correlation test. Test sizes of values in the heat map are proportional to the values in panel A. Labels on the left of the virus isolate list indicate the tier and clade of each isolate. Symbols in panel B are color-coded by vaccine groups as indicated, and *r* values that are bold and underlined are with raw_p values of <0.01 (Table 4).

Correlative relationships between antibody specificities and antibody function can provide insights on specificities that may mediate antiviral functions. We examined the association between linear antibody specificities and neutralization and ADCC responses measured at week 28 using data for all three groups. ID_50_ neutralizing titers for tier 1A and tier 1B clade B viruses were associated with binding magnitudes to peptides in the C1, V3, and the gp41_ID regions, with Spearman *r* values of >0.6 and raw_p values of <0.05 (Fig. 6 and Table 4). The highest Spearman *r* value was for the correlation between neutralizing activity for MN.3 and linear V3.1 binding (Spearman *r* = 0.85; false discovery rate [FDR]-adjusted *P* value [FDR_p] < 0.05). Interestingly, the highly conserved gp41_ID was among epitopes with the most scores above a Spearman *r* of 0.5 (raw_p < 0.05) for tier 1B and tier 2 viruses. The ADCC titers measured with B.63521Δ11 gp120-coated cells associated most strongly with two C1 epitopes (Spearman *r* value of 0.59 and 0.48 and raw_p of 0.027 and 0.08, respectively). Further examination of scatter plots revealed that correlations between binding magnitudes and neutralization titers for most pairs with *r* values of >0.6 were driven by data from all three groups, with the exception of MW965, for which correlations were driven by higher neutralization titers for the MVA+gp120 group (Fig. 6B).

**TABLE 4:**
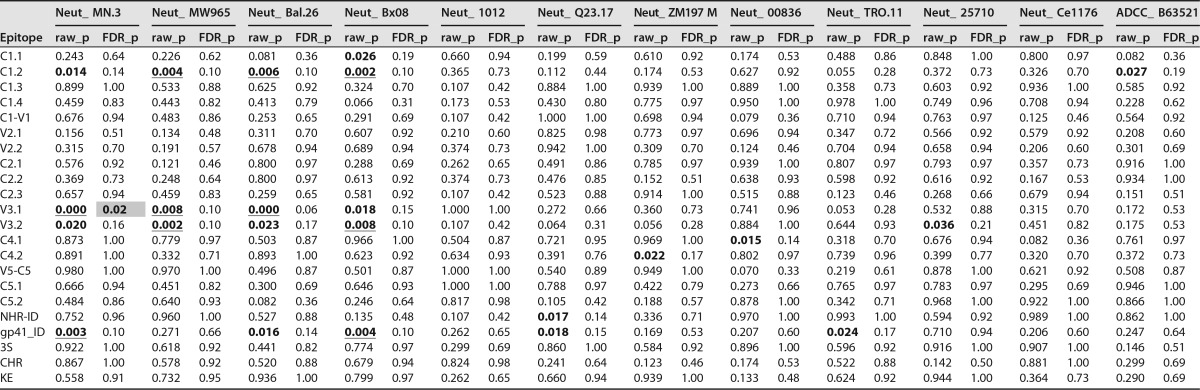
Raw *P* values and FDR-corrected (BH method) *P* values^*a*^ for Spearman correlation test in Fig. 6

a FDR_p values were obtained through multiple comparison correction of 264 spearman correlation tests in this table and 63 pair-wise comparisons Table 2 (across a total of 327 tests). Bold indicates raw_p values of <0.05, bold and underlining indicate raw_p values of <0.01, and bold and yellow highlight indicate FDR_p values of <0.05.

### T cell responses.

**FIG 7:**
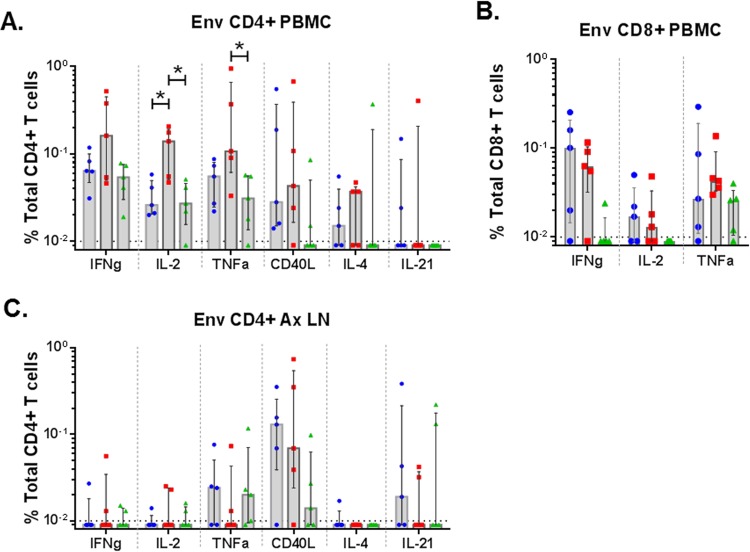
Env-specific T-cell responses. ICS was used to evaluate Env-specific CD4^+^ (A) and CD8^+^ (B) T cells in PBMC and Env-specific CD4^+^ T cells in axillary lymph nodes (C). Tests were done at 1 week following the 3rd boost. *, *P* < 0.05 for pairwise comparison (exact Wilcoxon rank sum test). All differences are insignificant once adjusted for multiple comparison (Table 2).

At 1 week following the last boost, lymphocytes were tested for effects of the boosts on Env-specific CD4^+^ T cells in blood and lymph nodes and on Env-specific CD8^+^ T cells in blood (Fig. 7). These assays, which tested overlapping peptide pools for stimulation of gamma interferon (IFN-γ), interleukin 2 (IL-2), tumor necrosis factor alpha (TNF-α), CD40L, IL-4, and IL-21, revealed higher proportions of elicited CD4^+^ T cells expressing IFN-γ, IL-2, TNF-α, and IL-4 in peripheral blood mononuclear cells (PBMC) than in lymph nodes but more similar levels of elicited CD40L-expressing cells in these two compartments (Fig. 7A and C). The MVA+gp120 group showed higher frequencies of IL-2- and TNF-α-expressing CD4^+^ T cells in PBMC than the other groups (*P* < 0.05, exact Wilcoxon rank sum) (Fig. 7A and Table 2). No significant difference was observed among groups for Env-specific CD8^+^ T cell response in PBMC (Fig. 7B).

## DISCUSSION

The current study compared the magnitude, durability, specificity, breadth, and functional activity of antibodies elicited by MVA+gp120 protein boosts, MVA+MVAgp140 boosts, or MVA-only boosts of a DNA-primed response. Our results reveal that the addition of either gp120 protein or MVA-expressed secreted gp140 to boosts elicits higher, broader, and more functional antibody responses than the MVA-only boosts. They also reveal that protein, but not MVAgp140, boosts enhanced CD4^+^ T cell responses. Below we discuss these results and how they might relate to protective vaccine responses.

The goal for adding boosts to the DNA/MVA vaccine was to enhance antibody responses to gp120, especially those that target the V1V2 region of gp120. Both B.63521 gp120 and MVAgp140 boosts enhanced these antibody responses. Following the 3rd and last boost, mean magnitudes of binding to the gp120 antigen panel and the V1V2 panel were 4.3- and 3.0-fold higher and 2.1- and 2.5-fold higher for the MVA+gp120 and MVA+MVAgp140 groups, respectively (Table 1). The gp120 vaccine immunogen boosts strongly enhanced the binding magnitude for the matched B.63521Δ11 protein (i.e., 17.9-fold by the gp120 protein boosts but only 3.5-fold by the heterologous MVAgp140 boosts [Fig. 1C and D]). The enhanced binding antibody response elicited by gp120 was more V3 focused, constituting 81% and 71% of total linear gp120 binding at the 2 time points tested, compared to 34 to 50% V3-specific IgG responses for the other 2 groups. No significant enhancement of V1V2 IgG responses with these immunogens was observed in the study. Further studies with other envelope immunogens are needed to determine the impact of immunogen sequence and antigenicity on the induction of V1V2 IgG responses.

In addition to the magnitude of binding antibody responses, the boost immunogens affected the durability of antibody responses and the performance of repeated boosts. The MVA+MVAgp140 group showed a trend for a slower decline and a more effective 3rd boost of responses than for the MVA+gp120 group. This lower contraction and higher boost with MVAgp140 suggest that MVAgp140 may elicit more durable and replenishable responses than the gp120 protein boost. Although lower levels of preboost antibody responses likely contributed to the efficiency of the 3rd MVAgp140 boost, there is precedent for late boosts, with MVA being highly effective in both preclinical and clinical settings (16–18).

In contrast to antibody responses, which were enhanced by the addition of both the gp120 protein and the MVA-expressed gp140 to boosts, only the gp120 boost increased the Env-specific CD4^+^ T cell response over those elicited by the MVA-only boost vaccine. The MVA-specific T cell response was not measured. However, we consider it unlikely that adding 1 × 10^8^ 50% tissue culture infective doses (TCID_50_) of MVA gp140 to the 1 × 10^8^ TCID_50_ of MVA62B in the MVA gp140 group significantly enhanced CD4^+^ responses because of the very shallow dose-response curve for MVA boosts of a constant DNA prime (19). In these studies, 100-fold increases in MVA dose were associated with only a 2- to 3-fold increase in elicited CD4^+^ T cells. In the canarypox prime/protein boost RV144 trial, the elicitation of five-function T cells expressing CD40L, IL-2, IL-4, IFN-γ, and TNF-α or three-function T cells expressing CD40L, IL-2, and IL-4 correlated with a reduced risk of infection (12). However, the elicitation of higher magnitudes of CD4^+^ T cells by the DNA prime/MVA+gp120 protein boost vaccine could also be a concern for HIV vaccine development because responding CD4^+^ T cells have been hypothesized to enhance infections (14, 20, 21). Successful prevention of virus acquisition may require a balance in antibody and CD4^+^ T cell responses in which changes in levels of virus-specific CCR5^+^ CD4^+^ T cells, preferential targets for HIV-1 infection (13), do not mute the effect of protective antibodies. Indeed, in 4 preclinical trials in which gp120 or gp140 protein boosts have been used with DNA/MVA prototypes of the GOVX-B11 vaccine, the protein boosts did not enhance protection but rather showed a trend for reduced prevention of virus acquisition or postinfection viremia control (22, 23; R. Amara, personal communication). In 3 of these trials, infection and/or poor control of viremia directly correlated with the induction of vaccine-specific IFN-γ^+^ CCR5^+^ CD4^+^ T cells in blood (22; Amara, personal communication). Thus, the ability of the MVAgp140 boosts to enhance antibody responses without boosting CD4^+^ T cell responses may be favorable for achieving protection by DNA/MVA vaccines and merits further investigation.

The gp120 and MVAgp140 boosts enhanced functional as well as binding antibody responses. The gp120 boosts enhanced both tier 1A neutralizing and ADCC responses, whereas MVAgp140 enhanced ADCC responses. Elicitation of higher neutralizing titers for tier 1A viruses by the protein boost likely reflected the strong ability of the gp120 protein to boost V3 responses.

Interestingly, for all three groups, the 2nd MVA boost elicited higher responses to tier 1B and tier 2 viruses than the 3rd boost. This phenomenon may reflect multiple protein boosts driving responses away from “neutralizing” conformational epitopes and toward “nonneutralizing” linear epitopes. It also could reflect immunoglobulin editing for autoreactive antibody responses, a phenomenon that can occur for tier 2 broadly neutralizing antibodies that are frequently autoreactive (24).

Both boost immunogens enhanced ADCC responses for bound B.63521Δ11 protein. This likely reflects both boosts enhancing the binding antibody response to B.63521Δ11. The higher ADCC responses elicited by MVA+gp120 than MVA+MVAgp140 likely reflected the very high titers of binding activity for B.63521Δ11mutC elicited by the matched gp120 boost.

To determine if any correlations existed between functional responses and responses to linear peptides, week 28 data from all groups were pooled and analyzed for correlations between neutralizing antibody and ADCC activity with binding to specific peptides. Neutralizing titers were found to associate with magnitude of binding to linear epitopes C1.1, C1.2, V3.1, V3.2, and gp41_ID (Spearman *r* > 0.6; raw_p < 0.05). These correlations were strongest and most frequent for the two tested tier 1 viruses. The finding of correlations with the gp41_ID region, albeit modest, are of interest because these correlations, with a highly conserved region in Env, included 4 of 9 tested tier 1B and tier 2 viruses. Monoclonal antibodies (MAbs) to the gp41_IDR capture virions (25, 26) and mediate ADCC (27) but have not been reported to have neutralizing activity. The anti-gp41_ID antibodies could also be biomarkers for a true mechanism that contributes to the neutralizing activities observed.

ADCC activity was found to be moderately associated with C1.1 and C1.2 binding. In RV144 subjects, a conformational C1 MAb (A32) blocked ADCC activity in over 90% of subjects (7). A32 did not bind to linear peptides in the microarray linear epitope mapping (data not shown). Whether the linear C1-binding antibodies, like the ones seen in this study, can mediate ADCC activity warrants further investigation. The higher linear C1-binding responses also may correlate with conformational C1 responses that were not quantified in this study.

In conclusion, gp120 protein and MVAgp140 were similar in their abilities to increase levels of antibody responses for gp120 and the V1V2 region of gp120 over the MVA-only boosts in the GOVX-B11 DNA/MVA vaccine regimen but differed in their abilities to enhance CD4^+^ T cell responses. These data demonstrate that both recombinant gp120 protein and MVA-expressed secreted gp140 are effective strategies for enhancing potential protective nonneutralizing antibody responses to HIV.

The study has had a direct impact on the protein boost protocol being advanced for the clade B DNA/MVA vaccine GOVX-B11. This study clearly demonstrated that under the conditions tested, B.63521Δ11 only modestly enhanced V1V2 responses. Because of this finding, both higher doses of B.63521Δ11 and a gp120 that is more effective at enhancing V1V2 responses are being tested in nonhuman primates and advanced to phase 1 clinical trials. Also, protein boosts have enhanced antibody responses but not protective responses in preclinical trials for DNA/MVA vaccines (see above). Understanding the nature of the enhanced CD4^+^ T cell response may allow identification of conditions under which protein boosts of DNA/MVA vaccines enhance both Ab and preclinical protective responses.

## MATERIALS AND METHODS

### Immunogens.

The GOVX-B11 vaccine consists of the pGA2/JS7 DNA and MVA/HIV62B (MVA62B) immunogens. The DNA prime expresses VLPs displaying the ADA gp160 Env. The DNA used in this trial also coexpressed rhesus granulocyte-macrophage colony-stimulating factor (GM-CSF) (5, 28). The DNA was produced, purified, and suspended in a proprietary buffer at 3 mg/ml at Aldevron (Fargo, ND). MVA62B expresses VLPs displaying ADA gp150 Env (29, 30). MVA62B (lot 001-03-12) was produced in chicken embryo fibroblasts (CEF) at IDT Biologica, purified by tangential-flow filtration, and suspended at 1 × 10^8^ TCID_50_ per ml in a proprietary buffer for storage and inoculation. MVA encodes a secreted ADA gp140 Env that was inserted into deletion II of MVA by plasmid insertion vector pLAS-2. It coexpresses the same HXB2/BH10 gag-pol as MVA62B. MVAgp140 was produced in CEF at the Laboratory for Viral Diseases, NIAID, NIH, purified by sucrose pad centrifugation, and suspended at 1 × 10^8^ TCID_50_ for storage and inoculation. B.63521Δ11mutC gp120 was produced by transient transfection at the Duke Human Vaccine Institute, Duke University. To prevent proteolytic clipping, mutations were introduced into the B.63521Δ11 gp120 V3 (SIR-GPGQT) region. Antigenicity analysis with a panel of MAbs showed similar affinities of binding of soluble CD4 (sCD4) and CD4 binding site broadly neutralizing antibodies (bnAbs) (VRC01 and CH103 MAbs) and for bnAbs for the V3 loop ASN332-centered oligomannose patch (PGT128, PGT125, and PGT121 MAbs) as well as the V3 loop MAb 19b, for B63521Δ11 with and without the *mutC* mutation (Alam et al., unpublished).

### Study design.

The animal study was conducted at Bioqual Inc., Rockville, MD, in accordance with all rules of the American Association for Laboratory Animal Care and under the supervision of the Institutional Animal Clinical Care and Use Committee. Three-year-old male rhesus macaques, prescreened for background for Env binding antibodies and activated T cells, were randomized into 3 study groups of 5 animals each by weight. For all groups, 3 mg of DNA was inoculated by needle and syringe into the upper left limb on weeks 0 and 8 and 1 × 10^8^ TCID_50_ of MVA62B into the upper left limb on weeks 18, 26, and 40. At the time of the MVA boosts, animals in group 2 also received 100 μg of B63521Δ11gp120 plus 500 μg of Alhydrogel (Brenntag Biosector; CAS 21645-51-2) and animals in group 3 also received 1 × 10^8^ TCID_50_ of MVAgp140, both delivered intramuscularly in 1 ml by needle and syringe to the upper right limb. Sera, PBMC, and rectal swabs were collected at regular intervals throughout the trial. Lymph nodes as well as an exsanguination bleed were collected at the end of the trial, 2 weeks following the last boost. The general health, weights, clinical blood counts (CBC), and clinical chemistries of animals were monitored throughout the trial.

### Binding antibody assays.

Binding antibody response was tested both in enzyme-linked-immunosorbent assays (ELISAs) and in binding antibody multiplex assays (BAMA). The ELISA uses Con6 gp120 produced in BHK cells (Duke) and gp41 produced in bacteria (NIH AIDS Reagent Program; catalog number 12027) as antigens for antibodies to gp120 and gp41, respectively. Assays were done using a standard curve of macaque IgG captured by a goat anti-rhesus antibody and results interpolated to estimate micrograms of specific IgG per ml as previously described (5). BAMA were conducted as previously described (4, 31, 32). Antibody titers (area under the curve [AUC]) were determined by serial dilutions of rhesus plasma (1:400, 5-fold) for the following antigens (provided by H. X. Liao and B. F. Haynes, Duke University, unless otherwise indicated): gp41 (subtype B) immunodiagnostics, group M consensus (ConSgp140CFI and Con6 gp120/B), and B.63521_Δ11gp120mutC (vaccine strain). The breadth of IgG response was characterized using a down-selected Ag panel developed by the Antigen Reagent Program (ARP) (Collaborative AIDS Vaccine Development-Collaborative AIDS Vaccine Immune Response Core) for consistently measuring binding antibody breadth in AIDS clinical trials (N. L. Yates, A. C. deCamp, B. T. Korber, H. X. Liao, A. Pinter, J. Peacock, L. H. Harris, S. Sawant, P. Hraber, X. Shen, S. Perks-Ngarm, P. Pitisuttithum, S. Nitayapan, P. W. Berman, M. L. Robb, G. Pantaleo, S. Zolla-Pazner, B. F. Haynes, S. M. Alam, D. C. Montefiori, and G. D. Tomaras, submitted for publication). This panel consists of 8 well-characterized gp120 HIV-1 envelope glycoprotein antigens and 8 oligomeric gp140 proteins (provided by H. X. Liao and B. F. Haynes, Duke University, unless otherwise indicated) and 16 gp70-V1/V2 scaffolds (provided by A. Pinter, State University of New Jersey). Plasma samples were tested at dilutions of 1:80 and 1:400 for binding-breadth BAMA. Mean fluorescence intensity (MFI) values within the linear range of the assay were used in breadth analysis. All assays were conducted under good clinical laboratory practice-compliant conditions. Positive controls included HIVIG and CH58 MAb titrations. Negative controls were blank beads, HIV-1-negative sera, and baseline (prevaccination) samples. To control for antigen performance, we used the preset criteria for each antigen that the positive-control titer for HIVIG, or for CH58 for V1V2 antigens, had to be within ±3 standard deviations of the mean tracked with a Levy-Jennings plot with preset acceptance of titer (calculated with a four-parameter logistic equation; SigmaPlot; Systat Software).

### Linear epitope mapping.

Serum epitope mapping of heterologous strains was performed as previously described (32–34), with minor modifications. Briefly, array slides were provided by JPT Peptide Technologies GmbH (Germany) by printing a library designed by B. Korber, Los Alamos National Laboratory, onto epoxy glass slides (PolyAn GmbH, Germany). The library contains overlapping peptides (15-mers overlapping by 12) covering 7 full-length gp160 consensus sequences (clades A, B, C, and D, group M, CRF1, and CRF2) and gp120 sequences of 6 vaccine strains (MN, A244, Th023, TV-1, ZM641, and 1086C). Three identical subarrays, each containing the full peptide library, were printed on each slide. All array slides were blocked with phosphate-buffered saline (PBS) containing 1% powdered milk, 5% normal goat serum, and 0.05% Tween for 1 h, followed by a 2-h incubation with 1:50 diluted test sera and a subsequent 45-min incubation with goat anti-human IgG conjugated with AF647 (Jackson ImmunoResearch, PA). Array slides were scanned at a wavelength of 635 nm with an InnoScan 710 AL scanner (Innopsys, Carbonne, France) using XDR mode. Images were analyzed using MagPix 8.0 software to obtain binding intensity values for all peptides. Binding of postimmunization serum to each peptide was subtracted from its baseline value, which was defined as the median signal intensity of the triplicates of the peptide for the matched prebleed serum plus 3 times the standard error of the triplicates. The magnitude of binding to each identified epitope was defined as the highest binding by a single peptide within the epitope region.

### Avidity.

The affinity maturation of the vaccine-elicited antibody responses to the immunodominant region of gp41 (gp41_ID) and V3 linear epitopes (V3.B and V3.C) was measured by an avidity index assay using the binding antibody multiplex assay. Avidity index was measured by inclusion of a denaturation step (treating the samples with 0.1 M sodium citrate, pH 3.0) (15). Untreated wells contained PBS during the avidity incubation step. The avidity index is defined as the MFI in the treated well divided by MFI in the untreated well times 100. Monoclonal antibodies 7B2 (35), 447-52D (36), and DH151 (37) were included as positive controls for gp41_ID, V3.B, and V3.C, respectively.

### ADCC-GranToxiLux assay.

Antibody dependent cellular cytotoxic activity mediated by test animal plasma/serum was detected according to our modification of the previously described GranToxiLux cell-mediated cytotoxicity procedure (38). CEM.NKRCCR5 target cells were coated with recombinant B.63521Δ11mutC gp120 protein. The assay readout is the percentage of antigen-coated target cells taking up granzyme B. A positive response is present if the peak percent granzyme B activity across 6 dilutions is greater than or equal to 8%.

### Neutralizing antibodies.

Neutralizing antibodies were measured as a function of reductions in luciferase (Luc) reporter gene expression after a single round of infection in TZM-bl cells (39, 40). TZM-bl cells (also called JC57BL-13) were obtained from the NIH AIDS Research and Reference Reagent Program, as contributed by John Kappes and Xiaoyun Wu. Briefly, a pretitrated dose of virus was incubated with serial 3-fold dilutions of test sample in duplicate in a total volume of 150 μl for 1 h at 37°C in 96-well flat-bottom culture plates. Freshly trypsinized cells (10,000 cells in 100 μl of growth medium containing 75 μg/ml of DEAE dextran) were added to each well. One set of 8 control wells received cells plus virus (virus control) and another set received cells only (background control). After 48 h of incubation, 100 μl of cells was transferred to a 96-well black solid plate (Costar) for measurements of luminescence using the Britelite luminescence reporter gene assay system (PerkinElmer Life Sciences). Assay stocks of molecularly cloned Env-pseudotyped viruses were prepared by transfection in 293T/17 cells (American Type Culture Collection) and titrated in TZM-bl cells as described previously (39). This assay has been formally optimized and validated (40) and was performed in compliance with good clinical laboratory practices, including participation in a formal proficiency testing program (41). Additional information on the assay and all supporting protocols may be found at http://www.hiv.lanl.gov/content/nab-reference-strains/html/home.htm.

### T cell responses.

HIV-specific cellular immune responses were assessed by multiparameter intracellular cytokine staining (ICS) assays following stimulation with GOVX-B11 vaccine-specific HIV peptides (15-mers overlapping by 11) as previously described (42). Panel one used anti-IFN-γ (clone B27), anti-IL-2 (clone MQ1-17H12), and anti-TNF-α (clone MAb11) antibodies, and a 2nd panel used anti-CD40L (clone TRAP1), anti-IL-4 (clone 8D4-8), and anti-IL-21 (clone 3A3-N2.1) antibodies. Both panels sorted cells using anti-CD3 (clone SP34-2), anti-CD4 (clone L200), and anti-CD8 (clone SK1) antibodies. All antibodies were obtained from BD Biosciences. Both panels also sorted live cells using a LIVE/DEAD fixable green stain kit (Thermo Fisher Scientific).

### Statistics.

Antibody and T cell response measurements were first screened in an omnibus test. Differences between pairs of groups in the levels of measurements that showed a *P* value of <0.05 were further tested using Wilcoxon rank sum exact test. Correlations between binding antibody responses and neutralization and ADCC responses were tested using the Spearman correlation test. *P* values from the Wilcoxon rank sum test and Spearman correlation test were corrected for false discovery rate (FDR) using the Benjamini-Hochberg (BH) method (43). Statistical significance indicated on figures are based on *P* values that are not controlled for multiple comparison (raw_p). Both raw_p and FDR-adjusted *P* values (FDR_p) are shown in Tables 2 and 4.

## References

[1] Goepfert PA, Elizaga ML, Seaton K, Tomaras GD, Montefiori DC, Sato A, Hural J, DeRosa SC, Kalams SA, McElrath MJ, Keefer MC, Baden LR, Lama JR, Sanchez J, Mulligan MJ, Buchbinder SP, Hammer SM, Koblin BA, Pensiero M, Butler C, Moss B, Robinson HL, HVTN 205 Study Group, National Institutes of Allergy and Infectious Diseases HIV Vaccines Trials Network 2014 Specificity and 6-month durability of immune responses induced by DNA and recombinant modified vaccinia Ankara vaccines expressing HIV-1 virus-like particles. J Infect Dis 210:99–110. doi:10.1093/infdis/jiu003. 24403557 PMC4072895

[2] Eroshkin AM, LeBlanc A, Weekes D, Post K, Li Z, Rajput A, Butera ST, Burton DR, Godzik A 2014 bNAber: database of broadly neutralizing HIV antibodies. Nucleic Acids Res 42:D1133–D1139. doi:10.1093/nar/gkt1083. 24214957 PMC3964981

[3] Zolla-Pazner S, deCamp A, Gilbert PB, Williams C, Yates NL, Williams WT, Howington R, Fong Y, Morris DE, Soderberg KA, Irene C, Reichman C, Pinter A, Parks R, Pitisuttithum P, Kaewkungwal J, Rerks-Ngarm S, Nitayaphan S, Andrews C, O'Connell RJ, Yang ZY, Nabel GJ, Kim JH, Michael NL, Montefiori DC, Liao HX, Haynes BF, Tomaras GD 2014 Vaccine-induced IgG antibodies to V1V2 regions of multiple HIV-1 subtypes correlate with decreased risk of HIV-1 infection. PLoS One 9:e87572. doi:10.1371/journal.pone.0087572. 24504509 PMC3913641

[4] Haynes BF, Gilbert PB, McElrath MJ, Zolla-Pazner S, Tomaras GD, Alam SM, Evans DT, Montefiori DC, Karnasuta C, Sutthent R, Liao HX, DeVico AL, Lewis GK, Williams C, Pinter A, Fong Y, Janes H, DeCamp A, Huang Y, Rao M, Billings E, Karasavvas N, Robb ML, Ngauy V, de Souza MS, Paris R, Ferrari G, Bailer RT, Soderberg KA, Andrews C, Berman PW, Frahm N, De Rosa SC, Alpert MD, Yates NL, Shen X, Koup RA, Pitisuttithum P, Kaewkungwal J, Nitayaphan S, Rerks-Ngarm S, Michael NL, Kim JH 2012 Immune-correlates analysis of an HIV-1 vaccine efficacy trial. N Engl J Med 366:1275–1286. doi:10.1056/NEJMoa1113425. 22475592 PMC3371689

[5] Smith JM, Amara RR, Campbell D, Xu Y, Patel M, Sharma S, Butera ST, Ellenberger DL, Yi H, Chennareddi L, Herndon JG, Wyatt LS, Montefiori D, Moss B, McClure HM, Robinson HL 2004 DNA/MVA vaccine for HIV type 1: effects of codon-optimization and the expression of aggregates or virus-like particles on the immunogenicity of the DNA prime. AIDS Res Hum Retroviruses 20:1335–1347. doi:10.1089/aid.2004.20.1335. 15650426

[6] Liao HX, Tsao CY, Alam SM, Muldoon M, Vandergrift N, Ma BJ, Lu X, Sutherland LL, Scearce RM, Bowman C, Parks R, Chen H, Blinn JH, Lapedes A, Watson S, Xia SM, Foulger A, Hahn BH, Shaw GM, Swanstrom R, Montefiori DC, Gao F, Haynes BF, Korber B 2013 Antigenicity and immunogenicity of transmitted/founder, consensus, and chronic envelope glycoproteins of human immunodeficiency virus type 1. J Virol 87:4185–4201. doi:10.1128/JVI.02297-12. 23365441 PMC3624376

[7] Bonsignori M, Pollara J, Moody MA, Alpert MD, Chen X, Hwang KK, Gilbert PB, Huang Y, Gurley TC, Kozink DM, Marshall DJ, Whitesides JF, Tsao CY, Kaewkungwal J, Nitayaphan S, Pitisuttithum P, Rerks-Ngarm S, Kim JH, Michael NL, Tomaras GD, Montefiori DC, Lewis GK, DeVico A, Evans DT, Ferrari G, Liao HX, Haynes BF 2012 Antibody-dependent cellular cytotoxicity-mediating antibodies from an HIV-1 vaccine efficacy trial target multiple epitopes and preferentially use the VH1 gene family. J Virol 86:11521–11532. doi:10.1128/JVI.01023-12. 22896626 PMC3486290

[8] Alam SM, Liao HX, Tomaras GD, Bonsignori M, Tsao CY, Hwang KK, Chen H, Lloyd KE, Bowman C, Sutherland L, Jeffries TL Jr, Kozink DM, Stewart S, Anasti K, Jaeger FH, Parks R, Yates NL, Overman RG, Sinangil F, Berman PW, Pitisuttithum P, Kaewkungwal J, Nitayaphan S, Karasavva N, Rerks-Ngarm S, Kim JH, Michael NL, Zolla-Pazner S, Santra S, Letvin NL, Harrison SC, Haynes BF 2013 Antigenicity and immunogenicity of RV144 vaccine AIDSVAX clade E envelope immunogen is enhanced by a gp120 N-terminal deletion. J Virol 87:1554–1568. doi:10.1128/JVI.00718-12. 23175357 PMC3554162

[9] Rerks-Ngarm S, Pitisuttithum P, Nitayaphan S, Kaewkungwal J, Chiu J, Paris R, Premsri N, Namwat C, de Souza M, Adams E, Benenson M, Gurunathan S, Tartaglia J, McNeil JG, Francis DP, Stablein D, Birx DL, Chunsuttiwat S, Khamboonruang C, Thongcharoen P, Robb ML, Michael NL, Kunasol P, Kim JH, MOPH-TAVEG Investigators 2009 Vaccination with ALVAC and AIDSVAX to prevent HIV-1 infection in Thailand. N Engl J Med 361:2209–2220. doi:10.1056/NEJMoa0908492. 19843557

[10] Goepfert PA, Elizaga ML, Sato A, Qin L, Cardinali M, Hay CM, Hural J, DeRosa SC, DeFawe OD, Tomaras GD, Montefiori DC, Xu Y, Lai L, Kalams SA, Baden LR, Frey SE, Blattner WA, Wyatt LS, Moss B, Robinson HL, National Institute of Allergy and Infectious Diseases HIV Vaccine Trials Network 2011 Phase 1 safety and immunogenicity testing of DNA and recombinant modified vaccinia Ankara vaccines expressing HIV-1 virus-like particles. J Infect Dis 203:610–619. doi:10.1093/infdis/jiq105. 21282192 PMC3072720

[11] Buchbinder SP, Grunenberg NA, Sanchez BJ, Seaton KE, Ferrari G, Moody MA, Frahm N, Montefiori DC, Hay CM, Goepfert PA, Baden LR, Robinson HL, Yu X, Gilbert PB, McElrath MJ, Huang Y, Tomaras GD, HIV Vaccine Trials Network (HVTN) 094 Study Group 2017 Immunogenicity of a novel clade B HIV-1 vaccine combination: results of phase 1 randomized placebo controlled trial of an HIV-1 GM-CSF-expressing DNA prime with a modified vaccinia Ankara vaccine boost in healthy HIV-1 uninfected adults. PLoS One 12:e0179597. doi:10.1371/journal.pone.0179597. 28727817 PMC5519050

[12] Lin L, Finak G, Ushey K, Seshadri C, Hawn TR, Frahm N, Scriba TJ, Mahomed H, Hanekom W, Bart P-A, Pantaleo G, Tomaras GD, Rerks-Ngarm S, Kaewkungwal J, Nitayaphan S, Pitisuttithum P, Michael NL, Kim JH, Robb ML, O'Connell RJ, Karasavvas N, Gilbert P, C De Rosa S, McElrath MJ, Gottardo R 2015 COMPASS identifies T-cell subsets correlated with clinical outcomes. Nat Biotechnol 33:610–616. doi:10.1038/nbt.3187. 26006008 PMC4569006

[13] Douek DC, Brenchley JM, Betts MR, Ambrozak DR, Hill BJ, Okamoto Y, Casazza JP, Kuruppu J, Kunstman K, Wolinsky S, Grossman Z, Dybul M, Oxenius A, Price DA, Connors M, Koup RA 2002 HIV preferentially infects HIV-specific CD4+ T cells. Nature 417:95–98. doi:10.1038/417095a. 11986671

[14] Fauci AS, Marovich MA, Dieffenbach CW, Hunter E, Buchbinder SP. 2014 Immune activation with HIV vaccines. Science 344:49–51. doi:10.1126/science.1250672. 24700849 PMC4414116

[15] Wei X, Liu X, Dobbs T, Kuehl D, Nkengasong JN, Hu DJ, Parekh BS 2010 Development of two avidity-based assays to detect recent HIV type 1 seroconversion using a multisubtype gp41 recombinant protein. AIDS Res Hum Retroviruses 26:61–71. doi:10.1089/aid.2009.0133. 20063992

[16] Chamcha V, Kannanganat S, Gangadhara S, Nabi R, Kozlowski PA, Montefiori DC, LaBranche CC, Wrammert J, Keele BF, Balachandran H, Sahu S, Lifton M, Santra S, Basu R, Moss B, Robinson HL, Amara RR 2016 Strong, but age-dependent, protection elicited by a DNA/modified vaccinia Ankara simian immunodeficiency virus vaccine. Open Forum Infect Dis doi:10.1093/ofid/ofw034. PMC4800464 27006959

[17] Joachim A, Munseri PJ, Nilsson C, Bakari M, Aboud S, Lyamuya EF, Tecleab T, Liakina V, Scarlatti G, Robb M, Earl P, Moss B, Wahren B, Mhalu F, Ferarri G, Sandstrom E, Biberfeld G 27 1 2017 Three-year durability of immune responses induced by HIV-DNA and HIV-modified vaccinia virus Ankara and effect of a late HIV-MVA boost in Tanzanian volunteers. AIDS Res Hum Retroviruses doi: 10.1089/AID.2016.0251. PMC5564012 28027665

[18] Nilsson C, Godoy-Ramirez K, Hejdeman B, Brave A, Gudmundsdotter L, Hallengard D, Currier JR, Wieczorek L, Hasselrot K, Earl PL, Polonis VR, Marovich MA, Robb ML, Sandstrom E, Wahren B, Biberfeld G 2014 Broad and potent cellular and humoral immune responses after a second late HIV-modified vaccinia virus Ankara vaccination in HIV-DNA-primed and HIV-modified vaccinia virus Ankara-boosted Swedish vaccinees. AIDS Res Hum Retroviruses 30:299–311. doi:10.1089/aid.2013.0149. 24090081 PMC3938943

[19] Liu J, Wyatt LS, Amara RR, Moss B, Robinson HL 2006 Studies on in vitro expression and in vivo immunogenicity of a recombinant MVA HIV vaccine. Vaccine 24:3332–3339. doi:10.1016/j.vaccine.2006.01.017. 16472543

[20] Fouts TR, Bagley K, Prado IJ, Bobb KL, Schwartz JA, Xu R, Zagursky RJ, Egan MA, Eldridge JH, LaBranche CC, Montefiori DC, Le Buanec H, Zagury D, Pal R, Pavlakis GN, Felber BK, Franchini G, Gordon S, Vaccari M, Lewis GK, DeVico AL, Gallo RC 2015 Balance of cellular and humoral immunity determines the level of protection by HIV vaccines in rhesus macaque models of HIV infection. Proc Natl Acad Sci U S A doi:10.1073/pnas.1423669112. PMC4352796 25681373

[21] Lewis GK, DeVico AL, Gallo RC 2014 Antibody persistence and T-cell balance: two key factors confronting HIV vaccine development. Proc Natl Acad Sci U S A 111:15614–15621. doi:10.1073/pnas.1413550111. 25349379 PMC4226080

[22] Iyer SS, Gangadhara S, Victor B, Gomez R, Basu R, Hong JJ, Labranche C, Montefiori DC, Villinger F, Moss B, Amara RR 2015 Codelivery of envelope protein in alum with MVA vaccine induces CXCR3-biased CXCR5+ and CXCR5- CD4 T cell responses in rhesus macaques. J Immunol 195:994–1005. doi:10.4049/jimmunol.1500083. 26116502 PMC4506863

[23] Buge SL, Ma HL, Amara RR, Wyatt LS, Earl PL, Villinger F, Montefiori DC, Staprans SI, Xu Y, Carter E, O'Neil SP, Herndon JG, Hill E, Moss B, Robinson HL, McNicholl JM 2003 Gp120-alum boosting of a Gag-Pol-Env DNA/MVA AIDS vaccine: poorer control of a pathogenic viral challenge. AIDS Res Hum Retroviruses 19:891–900. doi:10.1089/088922203322493067. 14585221

[24] Verkoczy L, Diaz M 2014 Autoreactivity in HIV-1 broadly neutralizing antibodies: implications for their function and induction by vaccination. Curr Opin HIV AIDS 9:224–234. doi:10.1097/COH.0000000000000049. 24714565 PMC4127310

[25] Burton DR, Hessell AJ, Keele BF, Klasse PJ, Ketas TA, Moldt B, Dunlop DC, Poignard P, Doyle LA, Cavacini L, Veazey RS, Moore JP 2011 Limited or no protection by weakly or nonneutralizing antibodies against vaginal SHIV challenge of macaques compared with a strongly neutralizing antibody. Proc Natl Acad Sci U S A 108:11181–11186. doi:10.1073/pnas.1103012108. 21690411 PMC3131343

[26] Liu P, Williams LD, Shen X, Bonsignori M, Vandergrift NA, Overman RG, Moody MA, Liao HX, Stieh DJ, McCotter KL, French AL, Hope TJ, Shattock R, Haynes BF, Tomaras GD 2014 Capacity for infectious HIV-1 virion capture differs by envelope antibody specificity. J Virol 88:5165–5170. doi:10.1128/JVI.03765-13. 24554654 PMC3993833

[27] Moog C, Dereuddre-Bosquet N, Teillaud JL, Biedma ME, Holl V, Van Ham G, Heyndrickx L, Van Dorsselaer A, Katinger D, Vcelar B, Zolla-Pazner S, Mangeot I, Kelly C, Shattock RJ, Le Grand R 2014 Protective effect of vaginal application of neutralizing and nonneutralizing inhibitory antibodies against vaginal SHIV challenge in macaques. Mucosal Immunol 7:46–56. doi:10.1038/mi.2013.23. 23591718

[28] Hellerstein M, Xu Y, Marino T, Lu S, Yi H, Wright ER, Robinson HL 2012 Co-expression of HIV-1 virus-like particles and granulocyte-macrophage colony stimulating factor by GEO-D03 DNA vaccine. Hum Vaccin Immunother 8:1654–1658. doi:10.4161/hv.21978. 23111169 PMC3601140

[29] Wyatt LS, Earl PL, Liu JY, Smith JM, Montefiori DC, Robinson HL, Moss B 2004 Multiprotein HIV type 1 clade B DNA and MVA vaccines: construction, expression, and immunogenicity in rodents of the MVA component. AIDS Res Hum Retroviruses 20:645–653. doi:10.1089/0889222041217428. 15242542

[30] Wyatt LS, Earl PL, Vogt J, Eller LA, Chandran D, Liu J, Robinson HL, Moss B 2008 Correlation of immunogenicities and in vitro expression levels of recombinant modified vaccinia virus Ankara HIV vaccines. Vaccine 26:486–493. doi:10.1016/j.vaccine.2007.11.036. 18155813 PMC2262837

[31] Yates NL, Liao HX, Fong Y, Decamp A, Vandergrift NA, Williams WT, Alam SM, Ferrari G, Yang ZY, Seaton KE, Berman PW, Alpert MD, Evans DT, O'Connell RJ, Francis D, Sinangil F, Lee C, Nitayaphan S, Rerks-Ngarm S, Kaewkungwal J, Pitisuttithum P, Tartaglia J, Pinter A, Zolla-Pazner S, Gilbert PB, Nabel GJ, Michael NL, Kim JH, Montefiori DC, Haynes BF, Tomaras GD 2014 Vaccine-induced Env V1-V2 IgG3 correlates with lower HIV-1 infection risk and declines soon after vaccination. Sci Transl Med 6: 228ra239. doi: 10.1126/scitranslmed.3007730. PMC4116665 24648342

[32] Tomaras GD, Binley JM, Gray ES, Crooks ET, Osawa K, Moore PL, Tumba N, Tong T, Shen X, Yates NL, Decker J, Wibmer CK, Gao F, Alam SM, Easterbrook P, Abdool Karim S, Kamanga G, Crump JA, Cohen M, Shaw GM, Mascola JR, Haynes BF, Montefiori DC, Morris L 2011 Polyclonal B cell responses to conserved neutralization epitopes in a subset of HIV-1-infected individuals. J Virol 85:11502–11519. doi:10.1128/JVI.05363-11. 21849452 PMC3194956

[33] Shen X, Duffy R, Howington R, Cope A, Sadagopal S, Park H, Pal R, Kwa S, Ding S, Yang OO, Fouda GG, Le Grand R, Bolton D, Esteban M, Phogat S, Roederer M, Amara RR, Picker LJ, Seder RA, McElrath MJ, Barnett S, Permar SR, Shattock R, DeVico AL, Felber BK, Pavlakis GN, Pantaleo G, Korber BT, Montefiori DC, Tomaras GD 2015 Vaccine-induced linear epitope-specific antibodies to simian immunodeficiency virus SIVmac239 envelope are distinct from those induced to the human immunodeficiency virus type 1 envelope in nonhuman primates. J Virol 89:8643–8650. doi:10.1128/JVI.03635-14. 26018159 PMC4524233

[34] Gottardo R, Bailer RT, Korber BT, Gnanakaran S, Phillips J, Shen X, Tomaras GD, Turk E, Imholte G, Eckler L, Wenschuh H, Zerweck J, Greene K, Gao H, Berman PW, Francis D, Sinangil F, Lee C, Nitayaphan S, Rerks-Ngarm S, Kaewkungwal J, Pitisuttithum P, Tartaglia J, Robb ML, Michael NL, Kim JH, Zolla-Pazner S, Haynes BF, Mascola JR, Self S, Gilbert P, Montefiori DC 2013 Plasma IgG to linear epitopes in the V2 and V3 regions of HIV-1 gp120 correlate with a reduced risk of infection in the RV144 vaccine efficacy trial. PLoS One 8:e75665. doi:10.1371/journal.pone.0075665. 24086607 PMC3784573

[35] Zhang W, Godillot AP, Wyatt R, Sodroski J, Chaiken I 2001 Antibody 17b binding at the coreceptor site weakens the kinetics of the interaction of envelope glycoprotein gp120 with CD4. Biochemistry 40:1662–1670. doi:10.1021/bi001397m. 11327825

[36] Gorny MK, Conley AJ, Karwowska S, Buchbinder A, Xu JY, Emini EA, Koenig S, Zolla-Pazner S 1992 Neutralization of diverse human immunodeficiency virus type 1 variants by an anti-V3 human monoclonal antibody. J Virol 66:7538–7542. 1433529 10.1128/jvi.66.12.7538-7542.1992 PMC240465

[37] Moody MA, Gao F, Gurley TC, Amos JD, Kumar A, Hora B, Marshall DJ, Whitesides JF, Xia SM, Parks R, Lloyd KE, Hwang KK, Lu X, Bonsignori M, Finzi A, Vandergrift NA, Alam SM, Ferrari G, Shen X, Tomaras GD, Kamanga G, Cohen MS, Sam NE, Kapiga S, Gray ES, Tumba NL, Morris L, Zolla-Pazner S, Gorny MK, Mascola JR, Hahn BH, Shaw GM, Sodroski JG, Liao HX, Montefiori DC, Hraber PT, Korber BT, Haynes BF 2015 Strain-specific V3 and CD4 binding site autologous HIV-1 neutralizing antibodies select neutralization-resistant viruses. Cell Host Microbe 18:354–362. doi:10.1016/j.chom.2015.08.006. 26355218 PMC4567706

[38] Pollara J, Hart L, Brewer F, Pickeral J, Packard BZ, Hoxie JA, Komoriya A, Ochsenbauer C, Kappes JC, Roederer M, Huang Y, Weinhold KJ, Tomaras GD, Haynes BF, Montefiori DC, Ferrari G 2011 High-throughput quantitative analysis of HIV-1 and SIV-specific ADCC-mediating antibody responses. Cytometry A 79:603–612. doi:10.1002/cyto.a.21084. 21735545 PMC3692008

[39] Montefiori DC 2009 Measuring HIV neutralization in a luciferase reporter gene assay. Methods Mol Biol 485:395–405. doi:10.1007/978-1-59745-170-3_26. 19020839

[40] Sarzotti-Kelsoe M, Bailer RT, Turk E, Lin CL, Bilska M, Greene KM, Gao H, Todd CA, Ozaki DA, Seaman MS, Mascola JR, Montefiori DC 2014 Optimization and validation of the TZM-bl assay for standardized assessments of neutralizing antibodies against HIV-1. J Immunol Methods 409:16. doi:10.1016/j.jim.2013.11.022. PMC4040342 24291345

[41] Todd CA, Greene KM, Yu X, Ozaki DA, Gao H, Huang Y, Wang M, Li G, Brown R, Wood B, D'Souza MP, Gilbert P, Montefiori DC, Sarzotti-Kelsoe M 2012 Development and implementation of an international proficiency testing program for a neutralizing antibody assay for HIV-1 in TZM-bl cells. J Immunol Methods 375:57–67. doi:10.1016/j.jim.2011.09.007. 21968254 PMC3332116

[42] Kwa S, Sadagopal S, Shen X, Hong JJ, Gangadhara S, Basu R, Victor B, Iyer SS, LaBranche CC, Montefiori DC, Tomaras GD, Villinger F, Moss B, Kozlowski PA, Amara RR 2015 CD40L-adjuvanted DNA/modified vaccinia virus Ankara simian immunodeficiency virus (SIV) vaccine enhances protection against neutralization-resistant mucosal SIV infection. J Virol 89:4690–4695. doi:10.1128/JVI.03527-14. 25653428 PMC4442387

[43] Benjamini Y, Hochberg Y 1995 Controlling the false discovery rate: a practical and powerful approach to multiple testing. J R Stat Soc Series B Stat Methodol 57:289–300.

